# Pattern of fixation explains atypical eye processing during observation of faces with direct or averted gaze in autism (results of the INFoR Cohort)

**DOI:** 10.1371/journal.pone.0334878

**Published:** 2025-11-17

**Authors:** Olena V. Bogdanova, Volodymyr B. Bogdanov, Etienne Guillaud, Charles Laidi, Josselin Houenou, Richard Delorme, Myriam Ly‑Le Moal, Marion Leboyer, Manuel Bouvard, Jean‑René Cazalets, Anouck Amestoy

**Affiliations:** 1 Centre hospitalier Charles-Perrens, Pôle Universitaire de Psychiatrie de l’enfant et de l’adolescent, Bordeaux Cedex, France; 2 University Bordeaux, CNRS, INCIA, UMR, Bordeaux, France; 3 Fondation FondaMental, Creteil, France; 4 AP-HP, Hopital Henri Mondor, Departement Medico-Universitaire de Psychiatrie et d’Addictologie (DMU IMPACT), Créteil, France; 5 University Paris Est Créteil, INSERM U955, IMRB, Translational Neuro-Psychiatry, Créteil, France; 6 CEA, Neurospin, Université Paris-Saclay, Gif-Sur-Yvette, France; 7 Child Mind Institute, Center for the Developing Brain, New York, New York, United States of America; 8 Child and Adolescent Psychiatry Department, Robert Debré Hospital, University of Paris Cité, Paris, France; 9 Human Genetics and Cognitive Functions Unit, Pasteur Institute Paris, Paris, France; 10 Institut Roche, Tour horizons- Bureau 18M3, Roche, Boulogne‑Billancourt, France; University of Missouri Columbia, UNITED STATES OF AMERICA

## Abstract

One of the most reliable early predictors of autism is atypical social attention, particularly attenuated eye gaze contact. As a part of the InFoR cohort, a multicentric French longitudinal study, 88 autistic participants and 56 participants without autism performed a gaze discrimination task using 28 static pictures of faces with either direct or averted gaze. We monitored eye fixation behavior during face picture observation and analyzed subsequent key-press responses. The eyes of faces with direct gaze attracted more fixations than those of faces with averted gaze. Autistic participants showed significantly reduced Eye Fixations Indexes (EFI; a parameter derived from the number of fixations on eyes of the image; it reflects participant’s strategy of face observation) and longer response times (RTs), strongly and negatively correlated with each other. A mediational analysis demonstrated that the influence of group on RTs was mainly driven by the EFI. The EFI was related to the number of anticipatory saccades obtained for basic oculomotor tasks. The RTs were related to scores of Attention Deficit Hyperactivity Disorder Rating Scale (ADHD-RS), Behavior Rating Inventory of Executive Function (BRIEF) and severity of autism as tested by the Social Responsiveness Scale (SRS-2), but not to the level of social anxiety. Altogether we demonstrate that the eye fixation index during face observation was associated with attentional control and influenced judgment response of participants, while the task performance is affected by a wider range of individual variables.

## Introduction

Autism spectrum disorders (ASD) are neurodevelopmental lifelong conditions that affect a large number of people worldwide [1,2]. The core symptoms of ASD, as defined by DSM-5 [3], include restricted and repetitive patterns of behavior and persistent deficits in social communication and interaction. Alterations in the perception of social information conveyed through another person’s eyes are considered one of the early behavioral signs observed in autism [4,5].

The eyes tend to be the primary focus of attention. This is because they provide rich social information, including emotional expressions and gaze direction, which are crucial for social interactions. The “eye region” typically draws the first and most fixations, especially the area around the eyes and eyebrows. Eye-tracking technology has been used extensively to study these fixation patterns. Such studies often reveal altered order and duration of fixations when participants look at faces under various conditions (e.g., when viewing faces with different expressions, lighting conditions, or familiarity) [6]. Individuals with ASD may show atypical patterns of eye fixation on faces [7], often spending less time looking at the eyes and more time focusing on other features, which can affect their social communication skills. However, studies using eye-tracking to explore face and gaze stimuli perception in autistic people have reported a variety of results [4,8,9]. Several researchers have attempted to generalize these findings, suggesting, among others, eye avoidance [5], aversion to direct gaze [10], a lack of preference for eyes [11], a lack of preference for direct gaze [12,13], and atypical patterns in face scanning strategies [7] in autistic people.

The origins and consequences of altered eye contact in autism are still unclear and the subject of debate. The observation of another person’s eyes evokes modified brain and physiological responses in autistic people [14,15]. Early excitatory/inhibitory imbalances in the brain [16], may impact the development of social orientation toward gaze from the early stages of life in autism [17]. Consequently, in autistic individuals, both cortical and subcortical circuits involved in face exploration may be impacted. In contrast to individuals with typical development who commonly employ a strategy of exploring unfamiliar faces by looking at both eyes, this pattern is not typically observed in autistic adults [7].

Several theoretical models attempt to explain the atypical gaze behavior in autism. Among others, the hypersensitivity model proposes that for individuals with autism, the need to focus on others’ eyes triggers heightened arousal, often resulting in discomfort and aversive reactions [15,18,19]. This is thought to be linked to overactivation of the amygdala, a brain region associated with emotional processing. Another theoretical concept, called the hyposensitivity model, suggests that autistic individuals may not prioritize social information, leading to reduced salience assigned to social stimuli [20]. The fast-track detector model [21] posits that subcortical regions like the superior colliculus play a key role in quickly orienting social attention via oculomotor mechanisms [22]. These subcortical processes are thought to underpin the typical saccadic patterns observed during face perception [7].

To explore several hypothetical factors associated with atypical eye contact in autism, the current paper analyzes data from the unique French large dataset obtained from the InFoR multicentric longitudinal cohort study. This dataset encompasses information from epidemiological, clinical, neuropsychological, biochemical, eye-tracking, and neuroimaging experiments [23–25]. In our experimental design, participants freely explored pictures of unfamiliar faces with averted or centered gazes and subsequently provided answers regarding the gaze direction of the presented face. To characterize the fixation pattern, reflecting automated, successive fixations on both eyes of a face, we introduced the Eye Fixation Index (EFI). This three level measure at the level of a trail captures whether a participant did not fixate on the eyes at all, fixated on only one eye, or successfully fixated on both eyes. Then the score is summed up across the trials. By using the EFI, we aimed to provide a more nuanced approach to assessing eye gaze behavior, enabling us to detect differences in social attention that might otherwise be overlooked with purely continuous measures. In our opinion, the EFI may have the potential as a biomarker, contributing to a better measurements of gaze behavior in autism. By capturing patterns of eye fixation, the EFI may offer a more specific profile of social attention, which could help clarify the mechanisms underlying reduced eye contact commonly observed in autism spectrum disorder.

We hypothesized that the strategy employed to explore the pictures would differ between conditions (averted *vs* direct gaze) and between groups of participants. We expected that the focus of visual attention on task-relevant elements of the image would be higher in typically developing (TD) participants and would define task performance. Subsequently, we investigated whether the participants’ key-press responses to gaze direction identification were influenced by the strategy used to examine the eye region of the displayed faces.

The attentional preference to the eye region and direct eye contact is observed from very early in life and is believed to be governed by an innate subcortical mechanism [14]. Previous results from this cohort [23] provided evidence for particularities in orienting, shifting, and disengaging visual attention among participants with ASD, which specifically impacted accuracy and rates of saccade errors. In these tasks, participants are instructed to maintain fixation on the central fixation point and, immediately after the appearance of the lateral target, make a saccade towards the target as quickly as possible. The increased number of anticipatory saccades, observed in the study of participants with ASD may be interpreted as a misbalance in attention engagement and disengagement [23]. Building on the same cohort, we explored whether there is a relationship between participants’ approach to exploring the face image and their ability to maintain attentional focus, as well as their levels of attentional and executive functioning assessed using the Attention Deficit Hyperactivity Disorder Rating Scale (ADHD-RS) and the Behavior Rating Inventory of Executive Function (BRIEF). Finally, we also assessed whether participants’ behavior was influenced by the severity of autism and the social nature of the stimuli by examining the relationship between the task-derived variables and scores on the Social Responsiveness Scale (SRS-2) and the Liebowitz Social Anxiety Scale.

## Materials and methods

### Participants

All participants were part of a study named InFoR, sponsored by Inserm (the French National Institute for Health), in collaboration with the Roche Institute for Research and Translational Medicine. This study received approval from the local Ethics Committee, known as the ‘Comité de Protection des Personnes,’ on October 12th, 2012, and was authorized by the French authorities (AFSSAPS B80738-70). All study participants provided their informed written consent to participate, in accordance with French ethical guidelines. For minor participants, written informed consents were obtained from parents. Inclusion visits have been achieved between March 2013 and January 2015. The present study included 88 autistic participants (16 women) and 56 participants without autism (17 women), referred to as the ASD and TD groups in the following text. Detailed demographic data can be found in **Table 1**.

**Table 1:** Demographic and clinical characteristics of participants with typical development, TD group (n = 56) and autistic participants, ASD group (n = 88), mean±SD.

group	typical development, TD group, n = 56	autistic participants, ASD group, n = 88
biological sex	39 males; 17 females 30% females	72 males; 16 females 18% females
age < 18 y.o.	10.7 ± 3.4; n = 27	11.7 ± 3.1 n = 56;
age>=18 y.o.	29.4 ± 8.4; n = 29	29.3 ± 9.1 n = 32;
IQ	108.4 ± 16.5; n = 55	103.4 ± 19.0; n = 88;
SRS, Total Scores	33.3 ± 25.8; n = 49	170.3 ± 6.1* n = 81;
ADHD-RS, Total Scores	6.3 ± 6.6; n = 49	23.1 ± 11.8* n = 77;
BRIEF-GEC scores	86.5 ± 18.7; n = 49	144.2 ± 30.5* n = 71;
LSAS, Total Scores	22.2 ± 18.4; n = 49	63.2 ± 32.4* n = 73;

*p < 0.01, in comparison with TD group; Mann-Whitney U test.

IQ, intellectual quotient; SRS, social responsiveness scale, total score; ADHD-RS, attention deficit hyperactivity disorder rating scale, total score; BRIEF-GEC, behavior rating inventory of executive function, global executive composite score; LSAS, Liebowitz social anxiety scale, total score.

The inclusion and exclusion criteria for all participants were the same as previously published for a study of oculomotor functions [23]. Participants younger than 6 or older than 56 years old were not included. Autistic children, adolescents, and adults received their diagnosis through the Autism Diagnostic Interview-Revised (ADI-R) [26] and the Autism Diagnostic Observation Schedule 2 (ADOS 2) [27], which were administered and rated by trained clinicians. The diagnoses were based on the DSM-5 criteria for ASD [3]. All autistic participants were considered high-functioning, as they were included in the study when their total IQ score was above 70. IQ was assessed using the Wechsler Adult Intelligence Scale (WAIS-III or WAIS-IV) or Wechsler Child Intelligence Scale (WISC-IV) [28,29]. Control participants were recruited through advertisements in the local press and met the following criteria: no diagnosed psychiatric disorder, no first-degree family history of schizophrenia, schizoaffective disorder, autism, or bipolar disorder, and no known genetic or neurological disorders.

### Clinical and psychological evaluations

The clinical evaluations included the Social Responsiveness Scale (SRS) [30], which consists of 65 items and assesses the social communication and social interaction dimensions of ASD. IQ was assessed using the Wechsler Adult Intelligence Scale (WAIS-III or WAIS-IV) or the Wechsler Child Intelligence Scale (WISC IV) [28,29].

Executive functions were evaluated with the Behavior Rating Inventory of Executive Function (BRIEF) [31]. The BRIEF clinical scales measured the intensity of difficulties related to the nine domains of executive functioning in daily life. It consists of equivalent Self-Report (for adults) and Informant Report Forms (for children), each having 75 items and provides a score reflecting ‘overall functioning’ known as the Global Executive Composite (GEC). Higher scores indicate a higher level of dysfunction in executive functioning.

Attention deficits and hyperactivity comorbidity was assessed using the Attention-Deficit/Hyperactivity Disorder Rating Scale (ADHD-RS IV), a questionnaire designed for individuals aged 4–18 years old and adults [32]. The ADHD RS-IV was completed by either the parents of children or the adult participants themselves and scored by a clinician. Higher scores indicate a greater incidence of attention deficits and hyperactivity symptoms.

Symptoms of social anxiety were assessed using The Liebowitz Social Anxiety Scale (LSAS) [33,34]. The LSAS is one of the most widely used measures of social anxiety in both the general population and clinical samples. This scale evaluates fear and avoidance behaviors in social interaction situations.

### The gaze direction discrimination task

The experiments took place in a dimly illuminated room, with participants seated 60 cm in front of a Tobii Pro TX300 eye-tracker (Tobii Technology, Stockholm, Sweden), which has a sampling rate of 300 Hz. Stimuli were displayed on the integrated 23“screen, with a resolution of 1920x1080. Calibration was performed before each task presentation, and participants’ head positions were fixed using a chinrest. Instructions were displayed on the screen and also verbally explained by the experimenter before the task.

**Fig 1:**
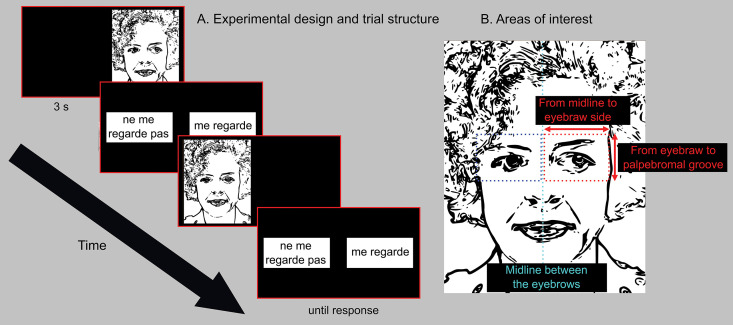
The stimuli and experimental paradigm. **A.** Experimental design with drawing representing stimuli image position and trial structure. 28 faces from 14 different individuals looking either directly or aside were presented for 3s each; after each face presentation a choice screen was presented with the option “Ne me regarde pas ” (does not look at me) and “me regarde” (looks at me). **B.** Definition of the Area Of Interest: Right and Left eyes.

The protocol comprised 28 trials, each involving a 3-second exploration of faces with either direct or averted gaze. After observing each face, participants had to indicate whether the gaze was direct or averted by pressing the keyboard keys as quickly as possible, as depicted in **Figure 1**. There was no time limitation for their responses. The entire task was completed in less than five minutes.

### Stimuli description

Three distinct sets of human faces (both genders) were selected from the same dataset used in the studies by Burra et al, 2017 and George et al, 2001 [35,36]. These sets consisted of pictures of 14 different individuals, encompassing both males and females, captured in front view with either direct gaze or averted gaze. Between trials, the direction of the averted gaze was counterbalanced between the left and right sides of the screen. There were no central fixation point in the trial. Each participant was presented with a total of 28 randomly chosen images. The images were randomly displayed on either the left or right side of the screen using TobiiStudio™ version 3.3.2 software (Tobii Technology, Stockholm, Sweden), during 3 seconds, followed by the participant’s unlimited time to make a response. The response options selected by pressing the ‘S’ or ‘L’ keys on the keyboard, were ‘does not look at me’ and ‘look at me’ (in French) (**Fig 1A**). The response keys were not counterbalanced across trials.

### Eye-tracking data preprocessing and variable definition

During the task, subjects were free to explore the presented picture. As the task of determining gaze direction required participants to focus on the person’s eyes, we defined the eyes of the image as the main area of interest (AOI) for our analysis (**Fig 1B**). The parameters of ocular fixations (X and Y positions in pixels, latency values, accuracy, inside/outside AOIs), and time stamps of the stimulus presentations were exported from TobiiStudio™. To define the eye fixations, we used a velocity-threshold identification filters in TobiiStudio™, following the recommendations of de Urabain et al. [37]. The parameters were set as follows: a maximum visual angle between fixations of 0.5 degrees, a minimum fixation duration of 60 ms, and a velocity threshold of 30 degrees per second. This filter is known for its conservative approach in detecting fixations, ensuring reliable fixation-saccade differentiation. We also conducted a data quality check, excluding eye-tracking data that showed a poor validity index (greater than 2) according to TobiiStudio™ internal criteria. During preprocessing, the percentage of rejected trials was calculated, resulting in an average of 5.25% (SD = 6.5%) of fixations being excluded due to this criterion. The cumulative number of fixations on the area of interest was selected as the most representative metric, as the fraction of fixations per trial does not provide meaningful information when values such as 0–3 fixations per trial are averaged. A limitation of this approach is that it does not account for trials with poor quality. However, as highlighted by our estimate, the entire face area was attended to in 78% of the trials, with every one of the 28 trials having at least some attention, and 95% of participants having at least 70% of their trials populated with face fixations. Unfortunately, the available database does not allow us to estimate the number of excluded trials, but this number appears to be low for the majority of the subjects. A custom-built toolbox developed with Matlab (version 2018a; The MathWorks, Inc., Natick, MA, USA) was used to compute the following eye fixation variables for each recorded trial. Using the Tobii algorithm, the eye fixation variables were estimated for the Areas of Interest (AOIs), which included the face, eyes, nose, and mouth of the presented image. The total fixation time on the entire screen was not estimated. To characterize eye fixations on the AOI of interest (eyes of the image), we examined the following variables:

[1] *Latency of the first fixation in the eye* (either left of right): the delay between the appearance of the image and the first fixation into the eye;
[2] *Total time of fixation*: the overall time spent looking into the eye(s) during the trial;
[3] *Number of fixations into the eyes*: the number of fixations in the eyes across all trials.

In addition to this, to better characterize the presence of automated pattern of AOI eye observation, we introduced a second-level qualitative variable, the *“eye fixation index, EFI”*.

For each trial, we attributed a value for that index which varied from 0 to 2, as follows: 0 – no fixation on eyes; 1 – at least one fixation on any of image’s eyes (left or right); 2 – at least one fixation on each eye. Therefore, for the whole task, the total EFI for each participant varied from 0 to 28.

For the task-related key press responses, we assessed *response accuracy* and *response times (RT)* in discriminating faces with direct or averted gaze. Response accuracy was determined as the percentage of all correct responses. We calculated the mean RT for averted and direct gaze trails for each participant. Total RTs for each participant were then calculated as the mean of RTs from all trials. One participant’s data were excluded from the analysis due to excessively long RT delay, exceeding 20 seconds.

*Oculomotor variables from basic oculomotor tasks.* We extracted data on the number of anticipatory saccades, which are eye movements not attributed to the appearance of the target stimuli from Step and Overlap oculomotor prosaccade tasks [23]. The experimental procedures, data analysis and results on the anticipatory saccades themselves were published before [23]. Here individual anticipatory saccade scores will be used only for correlational analysis.

### Statistical analysis

We conducted statistical analyses using MATLAB, Statisica software version 13.5.0.17 (TIBCO Inc.), and the open-source computer software JASP (Version 0.15, by JASP Team, 2021). To assess the normality of the data, we performed the Shapiro-Wilk normality test for all dependent variables. A resulting p-value of less than 0.05 confirmed that the data followed a non-parametric distribution. Consequently, for further analysis, we applied non-parametric statistics. We used the Mann-Whitney-Wilcoxon nonparametric test to assess group differences [38], and the Wilcoxon signed rank nonparametric test to evaluate the effect of the condition (averted or direct gaze of the image). For the effect size for the group, we calculated d of Cohen by subtracting the means and dividing the result by the pooled standard deviation. The general guidelines for interpreting the effect size are 0.2 = small effect; 0.5 = moderate effect and 0.8 = large effect. Cohen’s d was estimated along with Wilcoxon’s r effect size, which was based on the Z score from either the Wilcoxon or Mann-Whitney test divided by the square root of the sample size. The formula of the Wilcoxon effect size was taken from [39].

To investigate the causal relationships between groups, eye-tracking behavior and task-related key-press response, we conducted a mediational analysis. First, we normalized response times (RTs) and the eye fixation index using the rank-based inverse normal transformation with random tie-splitting [40]. To determine whether the inclusion of the *mediator variable*, EFI (M) explained a significant portion of the effect of *independent variable*, the group (X) on *outcome variable*, RT (Y), a Baron & Kenny’s 3-steps mediation analysis was then conducted [41] using The VBA toolbox [42] calculating: 1) regression of response Y (RT) on X (group), 2) regression of mediator M (EFI) on X (group) and 3) regression of Y (RT) on both X and M. The Sobel test [43] further estimated the significance of the reduction of relationship between X and Y when the mediator M was included. The effect size was reported in terms of adjusted percentage of variance explained.

We conducted partial rank order Spearman correlations between the response times (RTs), the Eye Fixation Index (EFI), and the clinical scores (ADHD-RS, BRIEF, SRS, LSAS) implemented in MATLAB, to disentangle the contribution of the group effect in these correlations. In order to correct for multitude of statistical tests we adjusted p values with a permutation-based control of Generalized Family-Wise Error Rate (GFWER) method [44,45]. Furthermore, we performed correlational analyses using the Spearman rank correlation method to examine the relationships between the EFI and anticipatory saccades percentage derived from the prosaccade tasks.

**Data Availability:** The final dataset and accompanying code are available on the Open Science Framework at https://osf.io/vs2gu/.

## Results

### Pattern of fixations

On *total fixation time* spent on AOI eyes we have found effect of group (Mann-Whitney-Wilcoxon nonparametric test, p < 0.05) with less time spent fixating on the image’s eyes by participants in ASD group then by participants in TD group, and effect of condition (Wilcoxon signed rank nonparametric test, p < 0.01, Cohen’s effect size d = 0.4093), with less time spent for averted gaze condition then for direct gaze, but no interaction (S1 Table).

For *latency of first fixation* on AOI eyes we observed effect of group (Mann-Whitney-Wilcoxon nonparametric test, p < 0.05, Cohen’s effect size d = 0.2729) with faster first fixation on the image’s eyes made by participants in TD group then by participants from ASD group, but neither effect of condition nor interaction (S2 Table).

We observed a main effect of the condition on *the total number of fixations* on the eyes of the image (Wilcoxon signed rank nonparametric test, p < 0.05). Specifically, participants fixated more on faces with direct gaze than on faces with averted gaze (S3 Table).

**Fig 2:**
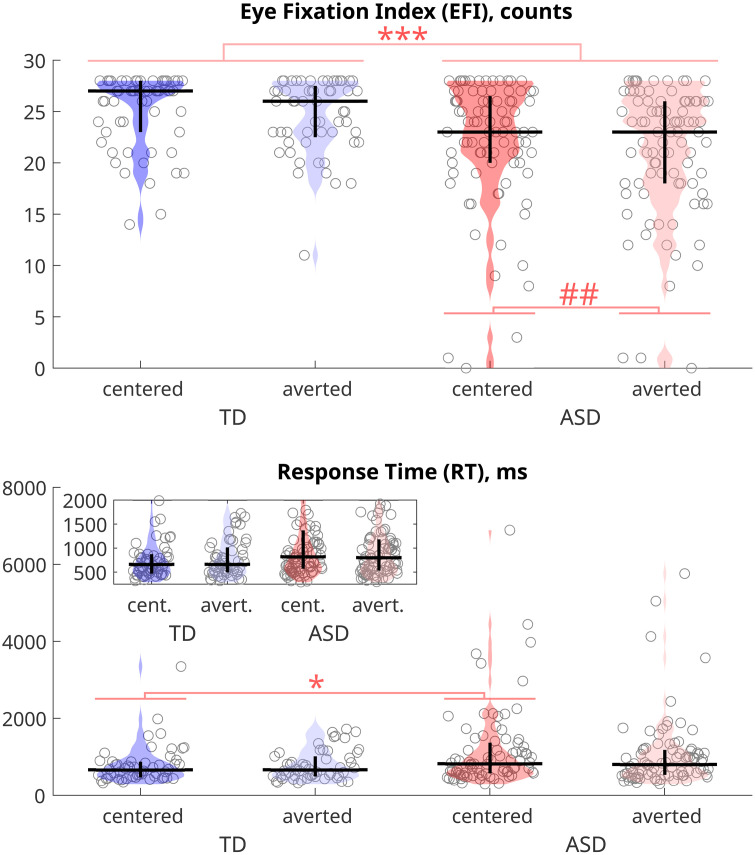
Effects of condition (centered or averted gaze) and group (TD or ASD) on Eye Fixation Index, EFI, counts, and Key-Press Response Time, RT, ms, during gaze discrimination task in direct and averted gaze conditions of participants in TD and ASD groups. The medians are shown with horizontal lines and inter-quartile intervals are shown with vertical lines. *** p < 0.001, * p < 0.05 for effect of the group (Mann-Whitney-Wilcoxon nonparametric test), ^###^ p < 0.001 for effect of the condition (Wilcoxon signed rank test).

The derivative of the number of fixations, the *Eye Fixation Index (EFI)*, emerged as the most sensitive oculomotor measure to both group and condition differences (Fig 2, S4 Table). The EFI was significantly influenced by both group (p < 0.0005, Cohen’s effect size d = 0.6212) and condition of the image presentation (p < 0.0005). Participants exhibited a higher EFI when looking at faces with direct gaze compared to faces with averted gaze. Participants in the TD group had a significantly higher EFI compared to autistic participants. Therefore, eye fixation index better explains atypical eye processing than other eye fixation parameters during observation of faces with direct or averted gaze.

### Task-related key press response behavior

Participants without autism exhibited significantly shorter mean response times, RTs (785 ms) compared to autistic participants (1107 ms; p < 0.05, Mann-Whitney-Wilcoxon nonparametric test), as illustrated in **Fig 2** and detailed in S5 Table. Additionally, participants without autism achieved significantly higher mean accuracy scores (0.98) compared to autistic participants (0.92, p < 0.05, Mann-Whitney-Wilcoxon nonparametric test), as presented in S6 Table. There was no significant effect of condition on either RTs or the accuracy of the response.

### Analysis of mediation

**Fig 3:**
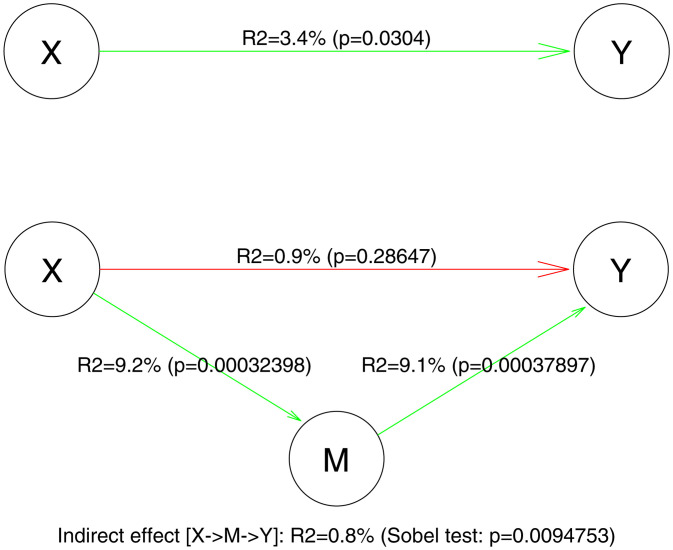
Results of the mediation analysis on three interrelated variables: the *independent variable* Group (X), the *mediator* eye fixation index (EFI, M) and the outcome variable Response Time (RT, Y).

Autistic participants exhibited reduced EFI and longer RTs compared to individuals in the TD group, and a significant negative correlation was found between these two parameters (Spearman R = −0.37; p = 0.000012, n = 136). To assess whether the differences in RTs and EFI between the two groups were independently influenced by group attribution or if RTs were mediated by the pattern of face exploration (EFI), we conducted a mediational analysis **(Fig 3).** A direct effect of group on RTs was confirmed (R2 = 3.4%, p = 0.03). However, when EFI was introduced as the mediator between the two variables, it weakened the direct relationship between the group and RT (R2 = 0.9%, p = 0.287), suggesting that the difference in RTs between the groups was primarily mediated by the pattern of face exploration (EFI).

### Relationship between EFI, RT, attentional control and executive functioning scores

To explore our secondary hypothesis that the observed group effects on eye behavior in the gaze direction judgment task might be linked to global atypicality in attentional control or executive functioning, we conducted a partial correlation analysis between EFI, RTs, and corresponding clinical scores (see S7 Tables). If corrected for group, no significant correlations were identified between EFI and ADHD-RS and BRIEF scores.

These clinical measures were highly influenced by group differences (see **Table 1**). Our analysis revealed that after controlling for the group effect, the positive relationships between RTs and higher ADHD-RS (R = 0.21, p < 0.05) and BRIEF (R = 0.33, p < 0.0005) total mean scores were significant. This suggests that the level of attentional and executive functioning may impact participants’ key-press behavioral responses in the gaze discrimination task independently of the group, whereas the relationship between EFI and clinical scores was group-driven because they disappeared after correction for group (see S7b and S7c Table).

We examined the relationship between RT, EFI, response accuracy and latency to look at the eyes, as these variables reflect the processing speed and strategies used for face observation (S8 Table). Our analysis shows that EFI was negatively correlated with latency (R = −0.52, p < 0.001) and positively correlated with accuracy (R = 0.28, p = 0.001). Additionally, RT was positively correlated with latency (R = 0.42, p < 0.001) and negatively correlated with accuracy (R = −0.42, p < 0.001). To further explore how gaze direction discrimination task performance is affected by attentional and executive functioning, we assessed relationships of RT and EFI with performance in visually guided saccade-based tasks. The parameters of performance reflect attentional engagement and disengagement processes and were previously analyzed within the same cohort. In these oculomotor tasks, the percentage of anticipatory saccades was found to be significantly higher for autistic participants (see Fig 3 in Amestoy et al., 2021, [23]. Using partial Spearman rank correlation for correction for group effect we discovered that participants who had lower EFI scores in the gaze direction discrimination task demonstrated greater overall likelihood of anticipatory saccades in STEP and OVERLAP conditions (R = −0.26, p = 0.004).

### Impacts of autism severity, social anxiety, age and gender

To assess whether participants’ performance in the gaze direction task was influenced by the severity of autism and/or social anxiety, we estimated partial correlation of their performance with the scores on the Social Responsiveness Scale (SRS-2) and the Liebowitz Social Anxiety Scale (LSAS). We identified that after controlling for the group effect, the positive relationships between RTs and higher SRS scores were significant (R = 0.26, p < 0.005) as well as negative relationship between EFI and SRS (R = −0.17). No significant correlation was found with social anxiety scores (S7 Table).

The age of participants exhibited strong correlations with RT (n = 136, R = −0.47, p < 1*10^−8^) and the accuracy of response (n = 137, R = 0.26, p = 0.002), and some relations with total fixation time (n = 142, R = 0.18, p < 0.05), and the latency of the first fixation (n = 142, R = −0.21, p < 0.05), but not with EFI. To explore other potential limitations, we evaluated the effect of gender on eye fixation and key-press responses by comparing groups (males vs. females) using the Mann-Whitney U test. However, we found no significant differences for any of the variables of interest.

## Discussion

The objective of the present study was to analyze how the patterns of exploration of faces with different gaze directions may vary between autistic participants and participants without autism. The absence of interaction between groups and the presentation condition indicates that both autistic and non-autistic individuals exhibit a similar reaction to the gaze direction of the presented face. However, autistic participants had diminished fixations on eyes overall, demonstrating a decreased interest in others’ eyes independent of the presentation condition.

Engaging in typical gaze processing, which involves fixating on both eyes, appears to improve key-press responses, as faster reaction times are associated with more consistent fixation behavior. Additionally, responses in the task were found to be correlated with scores related to executive and attentional control, as well as with basic oculomotor performance, highlighting the importance of visual attention engagement in the pattern of face observation.

### Effect of the gaze of the face on the image

We found that the condition of image presentation may influence the subject’s fixation on the image’s eyes: there were more fixations when the presented image had a direct gaze. This confirms that the automated pattern of eye fixations is influenced by the gaze direction of the observed face. Our data regarding the superiority of visual attention towards faces with direct gaze aligns with the concept of social attention governance, which postulates that faces with direct gazes should attract more fixations on the eye region due to the increased potential for social interaction. Our analysis does not allow us to confirm either hypersensitivity or hyposensitivity to *gaze direction* in autism. Consistent with our observations, more efficient visual scanning of faces with direct gaze compared to those with averted gaze was found for both autistics participants and their counterparts in the control group [46]. These results can also be explained by task instructions. When comparing the exploration patterns of the same image, Del Bianco et al. (2018) found that in tasks involving the judgement of gaze direction, autistic participants looked at the face image significantly longer compared to the other tasks such as free observation or object visual scanning [47].

### Eye fixation behavior and key press response in different participant groups

The analysis of common eye fixation variables (total fixation on eyes, latency of first fixation and total number of fixations) revealed lower values in autistic participants compared to their TD peers. The second-level variable, EFI, showed a significant decrease in autistic individuals and the largest effect size for between-group comparison. This suggests that EFI might be more effective in revealing differences between groups compared to the first-level variables. This effect is likely attributed to its focus on the dynamics of face exploration, including whether the eyes are meticulously observed one after another or not.

The relationship between EFI, RT and latency of first fixation remains highly significant even after controlling for the group factor (see S8 Table). This indicates that, in both groups, participants who looked at the eyes more quickly had more eye fixations and faster response times. Similarly, participants with greater EFI and faster RT were more accurate in their responses. Taken together, these findings may contribute to our understanding of the generalized dynamics in the processing of socially relevant information.

The significance of the *dynamics* of social stimuli processing has also been acknowledged [4]. During prolonged image observation, multiple “peaks” of increased attentional focus on social cues are observed [48,49]. However, autistic individuals do not exhibit these recurrent peaks; they demonstrate the same initial social engagement but fail to regain their attention after the decline of interest. In line with this, the distinct pattern of automatic face observation seen in typical development – where individuals consistently fixate on prominent facial features like eyes (one after another) and mouth [7] – no longer emerges in autism. This alteration may contribute to specific difficulties in processing and responding to social stimuli.

### Relevance of existing models for explaining group differences in task-related performances

The hypersensitivity model suggests that the need to look into others’ eyes or observe direct gaze triggers heightened arousal in autistic individuals, often resulting in discomfort and aversive reactions [15,18,19]. This has been linked to overactivation of the amygdala, a brain region associated with emotional salience. Notably, in autism, this heightened amygdala reactivity is observed only when tasks explicitly instruct participants to focus on the eyes [14]. However, our findings indicate that *gaze* hypersensitivity in ASD is distinct from *eyes* hypersensitivity. In our task, a significant difference between centered and averted gaze faces is observed only in the ASD group. Therefore, the reduced eye fixations observed for all images in the ASD group are unlikely to be explained by hypersensitivity to *gaze*. Rather, hypersensitivity to *eyes* may be responsible for this reduction in autistic participants.

The *hyposensitivity* model suggests that autistic individuals may not prioritize social information, leading to a reduced level of salience attributed to such stimuli [20]. In our study autistic participants had diminished fixations on eyes overall, demonstrating a decreased interest in others’ eyes independent of the presentation condition.

The *“fast-track detector”* model proposed by Senju and Johnson (2009) [21] suggests involvement of subcortical regions like the superior colliculus into rapid orientation of social attention via oculomotor mechanisms [22]. Such subcortical processes may underlie the typical saccadic pattern observed during face observation [7]. The decline in EFI during face observation in our task may be indicative of interruptions of such automated fixation pattern in autistic participants.

The lack of attentional engagement when observing socially relevant stimuli can result in subsequent variations in socially evoked responses. Consistent with this idea, we observed that individuals who had reduced EFI tended to have longer key-press RTs in the task. Furthermore, the results of the mediational analysis indicated that slower behavioral responses were influenced by decreased semi-voluntary eye fixations during the observation of face images. This aligns with other studies that have shown that the quantity of fixations on the eyes serves as a significant predictor for performance in social tasks, such as face or emotion recognition [50].

### Relationships between task-related measures, clinical scores and basic oculomotor behavior

The observed group effects on the eye fixation and key press behavior might be attributed to alterations in attentional, executive or oculomotor control functions previously documented in autistic individuals [23,51]. The ability of autistic participants to maintain focus of attention may be specifically impaired in case of social stimuli [52]. Here we found that participants’ key press responses are related to clinical scores characterizing executive functions and attentional deficits after accounting for group effect. However, the relationship between EFI and these clinical indexes is not significant in partial correlation analysis, indicating a relatively stronger between-group effect in gaze behaviors affecting both gaze behavior and task performance. EFI values were negatively related to the percentages of anticipatory saccades observed previously in visually guided saccade-based tasks, confirming that the ability to sustain attentional engagement may impact the eye fixation behavior during the observation of socially relevant stimuli particularly in autistic participants.

We found that autism severity, as estimated by SRS, was associated with slower decisions regarding gaze direction and decreased EFI. However, the relationship between SRS score and EFI appears to be more dependent on the group effect than between SRS and RTs. Surprisingly, we did not find any relationship between social anxiety scores and performance on the gaze discrimination task. This may possibly be attributed to the task’s diminished affective content, which included neutral expressions of the stimuli face, static images instead of dynamic videos, and the absence of an obligation to fixate on the presented face.

### Limitations

Due to the nature of the cohort study, our sample of participants is not perfectly balanced in terms of age and gender distribution. To assess the impacts of participants’ age on their performance, we correlated variables that showed group sensitivity and found a relationship with RTs, total number of eye fixation and latency of the first fixation but not EFI. In addition, we tested whether gender groups differ in their performance, but no significant results were found for any of the variables of interest. In our study design, face pictures were displayed on the left or right side of the presentation screen. Therefore, during trials with faces that had straight-ahead gazes, the gaze was not actually directed at the participant. It cannot be excluded that this might have influenced the responses of some participants. It is important to note that the calculation algorithm of EFI may possess a potential bias: a score of 10 can be obtained either because a participant looked at one eye in ten different trials or because they looked at both eyes in five trials. However, this bias does not undermine the efficacy of characterizing the participant’s interest in the image’s eyes. Despite the limitations of our study, it is worth noting that the observed relationship may provide insights into the treatment of social information processing not only in autistic people but also in a broader population.

## Conclusions

Evaluation of eye fixation in non-forced tasks, like free viewing, is considered the optimal eye-tracking approach for enhancing the accuracy and specificity of autism characterization. In such scenarios, semi-qualitative variables such as EFI might offer greater sensitivity in unveiling group-specific traits compared to primary-level eye-tracking metrics. The way individuals fixate the partner’s eyes plays a crucial role in making judgments during social interactions, such as gaze direction discrimination and shared attention. The reduced attentional engagement may contribute to the modification of the eye fixation patterns observed in autistic individuals. Quicker decision-making regarding gaze direction is associated with executive functions, attention/hyperactivity symptoms, and social skills, which go beyond the differences attributed solely to autism.

## Supporting information

S1 Table Total fixation time on AOI eyes, ms, mean, SD of mean, median and inter-quartile interval for images with direct and averted gazes of participants with typical development, TD group (n = 56) and autistic participants, ASD group (n = 86). *p < 0.05 for effect of the group ^##^p < 0.01 for effect of the condition.
(DOCX)

S2 Table Latency of first fixation on AOI eyes, ms, mean SD of mean, median and inter-quartile interval for images with direct and averted gazes of participants with typical development, TD group (n = 56) and autistic participants, ASD group (n = 86). *p < 0.05 for effect of the group
(DOCX)

S3 Table Total number of fixations on AOI eyes, mean, SD of mean, median and range for images with direct and averted gazes of participants with typical development, TD group (n = 56) and autistic participants, ASD group (n = 88). ^#^p < 0.05 for effect of the condition.
(DOCX)

S4 Table Eye Fixation Index, EFI, mean, SD of mean, median and inter-quartile interval for images with direct and averted gazes of participants with typical development, TD group (n = 56) and autistic participants, ASD group (n = 88). ***p < 0.001 for effect of the group ^###^p < 0.001 for effect of the condition.
(DOCX)

S5 Table Key-press response time, RT, ms mean, SD of mean, median and inter-quartile interval for images with direct and averted gazes of participants with typical development, TD group (n = 54) and autistic participants, ASD group (n = 82). *p < 0.05 for effect of the group.
(DOCX)

S6 Table Accuracy of key-press responses, mean, SD of mean, median and range for images with direct and averted gazes of participants with typical development, TD group (n = 54) and autistic participants, ASD group (n = 83). *p < 0.05 for effect of the group.
(DOCX)

S7 Table a. Partial correlation (Spearman rank correlation, corrected for *group* effect) of clinical scores with task-related variables: eye-fixation index and key-press response time. **b.** Partial correlation (Spearman rank correlation, corrected for *gender* effect) of clinical scores with task-related variables: eye-fixation index and key-press response time pa – GFWER adjusted level of significance. **c.** Partial correlation (Spearman rank correlation, corrected for *age* effect) of clinical scores with task-related variables: eye-fixation index and key-press response time pa – GFWER adjusted level of significance. **d.** Partial correlation (Spearman rank correlation, corrected for *group, gender and age* effect) of clinical scores with task-related variables: eye-fixation index and key-press response time.
(DOCX)

S8 Table Partial correlation (Spearman rank correlation, corrected for group, gender and age effect) of Latency of first fixation and Accuracy of key-press responses with task-related variables: eye-fixation index and key-press response time. (DOCX)
