# Supplementary material for: Pattern of fixation explains atypical eye processing during observation of faces with direct or averted gaze in autism (results of the INFoR Cohort)
Source: PLoS One. 2025 Nov 17;20(11):e0334878. doi: 10.1371/journal.pone.0334878 (PMC12622839; doi:10.1371/journal.pone.0334878)
Supplement: S1 Table — *p < 0.05 for effect of the group ##p < 0.01 for effect of the condition. (DOCX) [file pone.0334878.s001.docx]

For two populations when the observations are paired (two conditions) the Wilcoxon signed rank nonparametric test in MatLab was used; for two unpaired samples (effect of group) Mann-Whitney-Wilcoxon non parametric test in MatLab was applied.

Cardillo G. (2009). MWWTEST: Mann-Whitney-Wilcoxon nonparametric test for two unpaired samples. http://www.mathworks.com/matlabcentral/fileexchange/25830

On total fixation time spent on AOI eyes we have found effect of group (Mann-Whitney-Wilcoxon nonparametric test, p<0.05, Cohen d = 0.4093) with less time spent fixating on the image’s eyes by participants in ASD group then by participants in TD group, and effect of condition (Wilcoxon signed rank nonparametric test, p<0.01), with less time spent for averted gaze condition then for direct gaze, but no interaction (**S1 Table**).

**Effect size in the** **Wilcoxon signed-rank test**

The effect size indicates how large the observed effect is compared to the random noise. There are several measures to calculate the effect size in the Wilcoxon test. A common method is to use r, defined as:


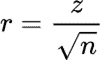


Where z is the standardized test statistic value from the Wilcoxon test and n is the total number of observations (i.e., the sum of the sizes of both groups).

The value of r can range from -1 to 1, with values near 0 indicating that there is no effect and values near -1 or 1 indicating a strong effect. The sign of r indicates the direction of the effect.

The following table can be used to interpret the effect size (effect size r according to Cohen (1988)).

| \|r\| < 0.1 | no effect / very small effect |
| --- | --- |
| \|r\| = 0.1 | small effect |
| \|r\| = 0.3 | medium effect |
| \|r\| = 0.5 | large effect |

**S1 Table. Total fixation time on AOI eyes, ms, mean, SD of mean, median and inter-quartile interval for images with direct and averted gazes of participants** **with typical development, TD group (n=56) and autistic participants, ASD group (n=86)** *p<0.05 for effect of the group ^##^p<0.01 for effect of the condition

|  | NT,  n=56 | ASD,  n=86 | all,  n=142 | p_gr | Coh. d | Wil. r |
| --- | --- | --- | --- | --- | --- | --- |
| cond 1 | 1757±497  1757[1453:2105] | 1516±588*  1564[1139:1907] | 1611±565  1664[1260:2042] | **0.013** | 0.44 | 0.21 |
| cond 2 | 1661±437 ^##^  1700[1434:1959] | 1478±553  1525[1091:1813] | 1550±516 ^##^  1605[1218:1903] | 0.051 | 0.37 | 0.16 |
| mean | 1709±451  1715[1483:2047] | 1497±557*  1546[1116:1842] | 1581±526  1636[1236:1946] | **0.022** | 0.42 | 0.19 |
| diff c2-c1 | -96±248  -95[-277:82] | -37±252  -19[-219:124] | -61±251  -52[-240:98] | 0.156 | 0.24 | 0.12 |
| p_cond | **0.006** | 0.222 | **0.007** |  |  |  |
| Coh. d | 0.39 | 0.15 | 0.24 |  |  |  |
| Wil. r | 0.37 | 0.13 | 0.23 |  |  |  |
