# Supplementary material for: Pattern of fixation explains atypical eye processing during observation of faces with direct or averted gaze in autism (results of the INFoR Cohort)
Source: PLoS One. 2025 Nov 17;20(11):e0334878. doi: 10.1371/journal.pone.0334878 (PMC12622839; doi:10.1371/journal.pone.0334878)
Supplement: S2 Table — *p < 0.05 for effect of the group (DOCX) [file pone.0334878.s002.docx]

For latency of first fixation on AOI eyes we observed effect of group (Mann-Whitney-Wilcoxon nonparametric test, p<0.05, Cohen d = 0.2729) with faster first fixation on the image’s eyes made by participants in TD group then by participants from ASD group, but neither effect of condition nor interaction **(S2 Table).**

**S2 Table. Latency of first fixation on AOI eyes, ms, mean SD of mean, median and inter-quartile interval for images with direct and averted gazes of participants with typical development, TD group (n=56) and autistic participants, ASD group (n=86)** *p<0.05 for effect of the group

|  | NT,  n=56 | ASD,  n=86 | all,  n=142 | p_gr | Coh. d | Wil. r |
| --- | --- | --- | --- | --- | --- | --- |
| cond 1 | 441±181  387[324:502] | 490±197  475[319:571] | 471±192  421[323:557] | 0.102 | 0.26 | 0.14 |
| cond 2 | 440±189  368[309:503] | 490±197*  442[366:565] | 470±195  415[326:548] | **0.026** | 0.26 | 0.19 |
| mean | 441±178  386[317:472] | 490±182*  457[367:554] | 470±182  440[335:535] | **0.024** | 0.27 | 0.19 |
| diff c2-c1 | -1±104  10[-58:61] | 0±149  2[-84:78] | -0±133  4[-69:73] | 0.872 | 0.01 | 0.01 |
| p_cond | 0.987 | 0.865 | 0.881 |  |  |  |
| Coh. d | 0.01 | 0.00 | 0.00 |  |  |  |
| Wil. r | 0.00 | 0.02 | 0.01 |  |  |  |
