# Supplementary material for: Pattern of fixation explains atypical eye processing during observation of faces with direct or averted gaze in autism (results of the INFoR Cohort)
Source: PLoS One. 2025 Nov 17;20(11):e0334878. doi: 10.1371/journal.pone.0334878 (PMC12622839; doi:10.1371/journal.pone.0334878)
Supplement: S3 Table — #p < 0.05 for effect of the condition. (DOCX) [file pone.0334878.s003.docx]

On total number of fixations on AOI eyes we have found effect of condition (Wilcoxon signed rank nonparametric test, p<0.05), with less number of fixations for averted gaze condition then for direct gaze, but nor effect of group (Cohen d = 0.1669) neither interaction **(S3 Table).**

**S3 Table. Total number of fixations on AOI eyes, mean, SD of mean, median and range for images with direct and averted gazes of participants with typical development, TD group (n=56) and autistic participants, ASD group (n=88)** ^#^p<0.05 for effect of the condition

|  | TD,  n=56 | ASD,  n=88 | all,  n=144 | p_gr | Coh. d | Wil. r |
| --- | --- | --- | --- | --- | --- | --- |
| cond 1 | 79.0±37.0  75.0[60.5:87.0] | 74.3±39.0  69.0[53.5:86.5] | 76.1±38.2  72.5[55.5:86.5] | 0.261 | 0.13 | 0.09 |
| cond 2 | 79.5±37.5  75.0[61.0:90.5] | 71.6±39.9^#^  67.0[47.5:83.0] | 74.6±39.0^#^  68.5[51.5:88.5] | 0.100 | 0.20 | 0.14 |
| mean | 79.3±36.8  74.0[62.3:87.5] | 72.9±38.8  67.8[52.0:87.8] | 75.4±38.0  70.5[54.8:87.5] | 0.172 | 0.17 | 0.11 |
| diff c2-c1 | 0.4±12.3  0.0[-6.0:4.5] | -2.7±13.7  -2.5[-10.5:3.0] | -1.5±13.2  -1.5[-9.5:4.0] | 0.085 | 0.24 | 0.14 |
| p_cond | 0.965 | **0.015** | **0.045** |  |  |  |
| Coh. d | 0.03 | 0.20 | 0.11 |  |  |  |
| Wil. r | 0.01 | 0.26 | 0.17 |  |  |  |
