# Supplementary material for: Pattern of fixation explains atypical eye processing during observation of faces with direct or averted gaze in autism (results of the INFoR Cohort)
Source: PLoS One. 2025 Nov 17;20(11):e0334878. doi: 10.1371/journal.pone.0334878 (PMC12622839; doi:10.1371/journal.pone.0334878)
Supplement: S4 Table — ***p < 0.001 for effect of the group ###p < 0.001 for effect of the condition. (DOCX) [file pone.0334878.s004.docx]

On eye fixation index we have found effect of group (Mann-Whitney-Wilcoxon nonparametric test, p<0.0005, Cohen d = 0.6212) with smaller EFI numbers observed for participants in ASD group then by participants in TD group, and effect of condition (Wilcoxon signed rank nonparametric test, p<0.0005), with less EFI numbers for averted gaze condition then for direct gaze, but no interaction (**S4 Table**).

**S4 Table. Eye Fixation Index, EFI, mean, SD of mean, median and inter-quartile interval for images with direct and averted gazes of participants with typical development, TD group (n=56) and autistic participants, ASD group (n=88)** ***p<0.001 for effect of the group ^###^p<0.001 for effect of the condition

|  | NT,  n=56 | ASD,  n=88 | all,  n=144 | p_gr | Coh. d | Wil. r |
| --- | --- | --- | --- | --- | --- | --- |
| cond 1 | 25.036±3.633  27.0[23:28] | 22.1±6.1***  23[20:26.5] | 23.2±5.4  25.0[21:27] | **0.001** | 0.59 | 0.28 |
| cond 2 | 24.536±3.567  26.0[22.5:27.5] | 21.1±6.3*** ^###^  23.0[18:26] | 22.5±5.6 ^###^  24.0[20:27] | **0.001** | 0.66 | 0.29 |
| mean | 24.786±3.4  26.0[23.3:27.5] | 21.6±6.0***  23.0[18.5:26.3] | 22.847±5.4  24.3[20.3:27.0] | **0.000** | 0.65 | 0.30 |
| diff c2-c1 | -0.500±2.264  0.0[-2.0:1.0] | -0.955±2.621  -1.0[-2.0:1.0] | -0.778±2.490  0.0[-2.0:1.0] | 0.324 | 0.19 | 0.08 |
| p_cond | 0.118 | **0.001** | **0.000** |  |  |  |
| Coh. d | 0.22 | 0.36 | 0.31 |  |  |  |
| Wil. r | 0.21 | 0.36 | 0.30 |  |  |  |
