# Supplementary material for: Pattern of fixation explains atypical eye processing during observation of faces with direct or averted gaze in autism (results of the INFoR Cohort)
Source: PLoS One. 2025 Nov 17;20(11):e0334878. doi: 10.1371/journal.pone.0334878 (PMC12622839; doi:10.1371/journal.pone.0334878)
Supplement: S5 Table — *p < 0.05 for effect of the group. (DOCX) [file pone.0334878.s005.docx]

On key-press response time we have found effect of group (Mann-Whitney-Wilcoxon nonparametric test, p<0.05) with faster responses found for participants in TD group then for participants in ASD group, but neither effect of condition nor interaction (**S5 Table**).

**S5 Table.** **Key-press response time, RT, ms mean, SD of mean, median and inter-quartile interval for images with direct and averted gazes of participants with typical development, TD group (n=54) and autistic participants, ASD group (n=82)** *p<0.05 for effect of the group

|  | NT,  n=54 | ASD,  n=82 | all,  n=136 | p_gr | Coh. d | Wil. r |
| --- | --- | --- | --- | --- | --- | --- |
| cond 1 | 782±495  662[472:879] | 1163±1052*  821[575:1373] | 1012±892  727[527:1118] | **0.014** | 0.46 | 0.21 |
| cond 2 | 787±388  663[497:1019] | 1051±942  794[534:1184] | 946±780  723[514:1116] | 0.146 | 0.37 | 0.12 |
| mean | 785±415  669[494:960] | 1107±956*  857[544:1351] | 979±801  766[520:1153] | **0.035** | 0.44 | 0.18 |
| diff c2-c1 | 5±316  3[-64:45] | -113±579  -27[-197:71] | -66±494  -8[-101:63] | 0.262 | 0.25 | 0.10 |
| p_cond | 0.773 | 0.093 | 0.159 |  |  |  |
| Coh. d | 0.01 | 0.19 | 0.13 |  |  |  |
| Wil. r | 0.04 | 0.19 | 0.12 |  |  |  |
