# Supplementary material for: Pattern of fixation explains atypical eye processing during observation of faces with direct or averted gaze in autism (results of the INFoR Cohort)
Source: PLoS One. 2025 Nov 17;20(11):e0334878. doi: 10.1371/journal.pone.0334878 (PMC12622839; doi:10.1371/journal.pone.0334878)
Supplement: S6 Table — *p < 0.05 for effect of the group. (DOCX) [file pone.0334878.s006.docx]

On accuracy of key-press responses we have found effect of group (Mann-Whitney-Wilcoxon nonparametric test, p<0.05) with more correct responses found for participants in TD group then for participants in ASD group, but neither effect of condition nor interaction (**S6 Table**).

**S6 Table.** **Accuracy of key-press responses, mean, SD of mean, median and range for images with direct and averted gazes of participants with typical development, TD group (n=54) and autistic participants, ASD group (n=83)** *p<0.05 for effect of the group

|  | TD,  n=54 | ASD,  n=83 | all,  n=137 | p_gr | Coh. d | Wil. r |
| --- | --- | --- | --- | --- | --- | --- |
| cond 1 | 0.98±0.04  1.00[1.00:1.00] | 0.92±0.18  1.00[0.93:1.00] | 0.95±0.15  1.00[0.93:1.00] | 0.149 | 0.44 | 0.12 |
| cond 2 | 0.99±0.03  1.00[1.00:1.00] | 0.91±0.21*  1.00[0.93:1.00] | 0.94±0.17  1.00[1.00:1.00] | **0.032** | 0.48 | 0.18 |
| mean | 0.98±0.03  1.00[0.96:1.00] | 0.92±0.18*  1.00[0.93:1.00] | 0.94±0.15  1.00[0.96:1.00] | **0.038** | 0.50 | 0.18 |
| diff c2-c1 | 0.01±0.05  0.00[0.00:0.00] | -0.01±0.14  0.00[0.00:0.00] | -0.00±0.12  0.00[0.00:0.00] | 0.798 | 0.14 | 0.02 |
| p_cond | 0.379 | 0.913 | 0.576 |  |  |  |
| Coh. d | 0.14 | 0.06 | 0.02 |  |  |  |
| Wil. r | 0.00 | 0.01 | 0.05 |  |  |  |
