# Supplementary material for: Pattern of fixation explains atypical eye processing during observation of faces with direct or averted gaze in autism (results of the INFoR Cohort)
Source: PLoS One. 2025 Nov 17;20(11):e0334878. doi: 10.1371/journal.pone.0334878 (PMC12622839; doi:10.1371/journal.pone.0334878)
Supplement: S7 Table — b. Partial correlation (Spearman rank correlation, corrected for gender effect) of clinical scores with task-related variables: eye-fixation index and key-press response time pa – GFWER adjusted level of significance. c. Partial correlation (Spearman rank correlation, corrected for age effect) of clinical scores with task-related variables: eye-fixation index and key-press response time pa – GFWER adjusted level of significance. d. Partial correlation (Spearman rank correlation, corrected for group, gender and age effect) of clinical scores with task-related variables: eye-fixation index and key-press response time. (DOCX) [file pone.0334878.s007.docx]

| **S7a Table. Partial correlation (Spearman rank correlation, corrected for *group* effect) of clinical scores with task-related variables: eye-fixation index and key-press response time** | | | | |
| --- | --- | --- | --- | --- |
|  | SRS | ADHD-RS | BRIEF | LSAS |
| **Eye Fixation index** |  |  |  |  |
| R2 *partial* Spearman rank  correlation, controlled for effect of group | **n=130**    **R=-0.17**    **p=0.058**  **pa = 0.015** | n=126    R=0.03    p=0.745 | n=120    R=-0.08    p=0.408 | n=122    R=0.02    p=0.824 |
| **Response time** |  |  |  |  |
| R2 *partial* Spearman rank  correlation, controlled for effect of group | **n=123**    **R=0.26**    **p=0.004**  **pa = 0.006** | **n=120**    **R=0.21**    **p=0.021**  **pa= 0.011** | **n=114**    **R=0.33**    **p=0.000**  **pa = 0** | n=116    R=-0.09    p=0.324 |

pa – GFWER adjusted level of significance

| **S7b Table. Partial correlation (Spearman rank correlation, corrected for *gender* effect) of clinical scores with task-related variables: eye-fixation index and key-press response time** pa – GFWER adjusted level of significance | | | | |
| --- | --- | --- | --- | --- |
|  | SRS | ADHD-RS | BRIEF | LSAS |
| **Eye Fixation index** |  |  |  |  |
| R2 *partial* Spearman rank  correlation, controlled for effect of group | **n=130**    **R=-0.34**    **p=0.000**  **pa = 0** | **n=126**  **R=-0.21**    **p=0.020**  **pa <0.001** | **n=120**    **R=-0.29**    **p=0.002**  **pa = 0.001** | n=122    R=-0.18  p=0.055 |
| **Response time** |  |  |  |  |
| R2 *partial* Spearman rank  correlation, controlled for effect of group | **n=123**    **R=0.28**    **p=0.002**  **pa <0.001** | **n=120**    **R=0.29**    **p=0.001**  **pa <0.001** | **n=114**    **R=0.37**    **p=0.000**  **pa = 0.001** | n=116    R=0.03    p=0.775 |

| **S7c Table. Partial correlation (Spearman rank correlation, corrected for *age* effect) of clinical scores with task-related variables: eye-fixation index and key-press response time** pa – GFWER adjusted level of significance | | | | |
| --- | --- | --- | --- | --- |
|  | SRS | ADHD-RS | BRIEF | LSAS |
| **Eye Fixation index** |  |  |  |  |
| R2 *partial* Spearman rank  correlation, controlled for effect of gender | **n=130**    **R=-0.33**    **p=0.000**  **pa = 0** | **n=126**    **R=-0.19**    **p=0.038**  **pa = 0** | **n=120**    **R=-0.27**    **p=0.003**  **p<0.001** | **n=122**    **R=-0.21**    **p=0.019**  **p = 0.002** |
| **Response time** |  |  |  |  |
| R2 *partial* Spearman rank  correlation, controlled for effect of gender | **n=123**    **R=0.28**    **p=0.002**  **pa = 0.002** | **n=120**    **R=0.19**    **p=0.035**  **pa<0.001** | **n=114**    **R=0.24**    **p=0.010**  **pa<0.001** | **n=116**    **R=0.20**    **p=0.036**  **pa <0.001** |

| **S7d Table. Partial correlation (Spearman rank correlation, corrected for *group, gender and age* effect) of clinical scores with task-related variables: eye-fixation index and key-press response time** | | | | |
| --- | --- | --- | --- | --- |
|  | SRS | ADHD-RS | BRIEF | LSAS |
| **Eye Fixation index** |  |  |  |  |
| R2 *partial* Spearman rank  correlation, controlled for effects of three factors | n=130    R=-0.16    p=0.074 | n=126    R=0.06    p=0.544 | n=120    R=-0.04    p=0.700 | n=122    R=-0.02    p=0.823 |
| **Response time** |  |  |  |  |
| R2 *partial* Spearman rank  correlation, controlled for effects of three factors | n=123    R=0.21    p=0.021 | n=120    R=0.11    p=0.253 | n=114    R=0.12    p=0.221 | n=116    R=0.07    p=0.434 |
