# Supplementary material for: Pattern of fixation explains atypical eye processing during observation of faces with direct or averted gaze in autism (results of the INFoR Cohort)
Source: PLoS One. 2025 Nov 17;20(11):e0334878. doi: 10.1371/journal.pone.0334878 (PMC12622839; doi:10.1371/journal.pone.0334878)
Supplement: S8 Table — (DOCX) [file pone.0334878.s008.docx]

| **S8 Table. Partial correlation (Spearman rank correlation, corrected for group, gender and age effect) of Latency of first fixation and Accuracy of key-press responses with task-related variables: eye-fixation index and key-press response time.** | | |
| --- | --- | --- |
|  | **Latency of first fixation** | **Accuracy of key-press responses** |
| **Eye Fixation index** |  |  |
| R2 *partial* Spearman rank  correlation, controlled for effects of three factors | n=142  R=-0.52  p=0.000 | n=137  R=0.28  p=0.001 |
| **Response time** |  |  |
| R2 *partial* Spearman rank  correlation, controlled for effects of three factors | n=134  R=0.42  p=0.000 | n=136  R=-0.42  p=0.000 |
